# Induction of ebolavirus cross-species immunity using retrovirus-like particles bearing the Ebola virus glycoprotein lacking the mucin-like domain

**DOI:** 10.1186/1743-422X-9-32

**Published:** 2012-01-25

**Authors:** Wu Ou, Josie Delisle, Jerome Jacques, Joanna Shih, Graeme Price, Jens H Kuhn, Vivian Wang, Daniela Verthelyi, Gerardo Kaplan, Carolyn A Wilson

**Affiliations:** 1 Division of Cellular and Gene Therapies, Center for Biologics Evaluation and Research, Bldg. 29B, Room 5NN22, 8800 Rockville Pike, Bethesda, MD 20892, USA; 2 Division of Emerging and Transfusion Transmitted Diseases, Center for Biologics Evaluation and Research FDA, Bethesda, MD, USA; 3 Biometric Research Branch, National Cancer Institute, Rockville, MD, USA; 4 NIH/NIAID, Integrated Research Facility at Fort Detrick, Frederick, MD, USA; 5 Division of Therapeutic Proteins, Office of Biotechnology Products, Center for Drug Evaluation and Review, FDA, Bethesda, MD, USA

**Keywords:** Ebola, Ebolavirus, Envelope glycoprotein, Filovirus, Mucin-like domain, Retrovirus, Virus-like particles, DNA vaccine

## Abstract

**Background:**

The genus *Ebolavirus *includes five distinct viruses. Four of these viruses cause hemorrhagic fever in humans. Currently there are no licensed vaccines for any of them; however, several vaccines are under development. Ebola virus envelope glycoprotein (GP_1,2_) is highly immunogenic, but antibodies frequently arise against its least conserved mucin-like domain (MLD). We hypothesized that immunization with MLD-deleted GP_1,2 _(GPΔMLD) would induce cross-species immunity by making more conserved regions accessible to the immune system.

**Methods:**

To test this hypothesis, mice were immunized with retrovirus-like particles (retroVLPs) bearing Ebola virus GPΔMLD, DNA plasmids (plasmo-retroVLP) that can produce such retroVLPs *in vivo*, or plasmo-retroVLP followed by retroVLPs.

**Results:**

Cross-species neutralizing antibody and GP_1,2_-specific cellular immune responses were successfully induced.

**Conclusion:**

Our findings suggest that GPΔMLD presented through retroVLPs may provide a strategy for development of a vaccine against multiple ebolaviruses. Similar vaccination strategies may be adopted for other viruses whose envelope proteins contain highly variable regions that may mask more conserved domains from the immune system.

## Background

The genus *Ebolavirus *is a member of the family *Filoviridae*. *Ebolavirus *includes five species: *Zaire ebolavirus *(Ebola virus, EBOV), *Sudan ebolavirus *(Sudan virus, SUDV), *Taï Forest ebolavirus *(Taï Forest virus, TAFV), *Reston ebolavirus *(Reston virus, RESTV), and *Bundibugyo ebolavirus *(Bundibugyo virus, BDBV) [1]. Except for RESTV, the ebolaviruses cause viral hemorrhagic fever (VHF) in humans. In particular, EBOV infection causes lethality up to 90% [2,3]. Other than supportive care, there is no FDA-approved treatment or vaccine for ebolavirus infections.

Ebolaviruses have been categorized by NIH/NIAID as Category A Priority Pathogens because they could be misused for the development of biological weapons. The availability of a vaccine that provides cross-protection against different ebolaviruses is essential for preparedness against natural outbreaks and acts of bioterrorism. While there has been progress in recent years towards development of ebolavirus vaccines, most vaccine candidates are based on antigens from one or two ebolaviruses only. Though some vaccine candidates have demonstrated evidence of cross-protection, many induce species-specific immune responses and protection [4-6].

The viral envelope glycoprotein GP_1,2 _is either a component of, or the sole viral antigen in many ebolavirus candidate vaccines. GP_1,2 _is presented on the surface of virions as trimers of GP_1_-GP_2 _heterodimers that are linked together through a disulfide bond [7]. The C-terminal region of GP_1_, designated as the mucin-like domain (MLD), is highly variable among different ebolaviruses and is highly *N*- and *O*-glycosylated. The MLD is thought to form a "glycan cap" that is hypothesized to prevent antibody binding to those epitopes shielded from recognition by the immune system, suggesting that the MLD with its glycan cap provides a mechanism of immune evasion [8-10]. In addition, the MLD-glycan cap appears to be a target for antibody responses to ebolaviruses, and may thus also serve as a decoy to divert an antibody response to the more conserved regions of the envelope [11-14]. The MLD is dispensable for GP_1,2_-mediated virus entry [7,15-17], and there appear to be no other known functions for the MLD other than immune shielding/evasion. We hypothesized that deletion of the MLD would expose the more conserved regions of GP_1,2_, such as the receptor-binding site [8,15,18,19], and induce an immune response to these more conserved regions that may result in cross-species immunity.

Virus-like particles (VLPs) are ideal immunogens because 1) they mimic wild-type pathogens in morphology and thus display antigens in their native conformations; 2) the particle size allows for efficient uptake by antigen presenting cells; and 3) presentation of the multimeric form of antigens on VLPs may cross-link B cell receptors and provide a strong stimulation signal [20,21]. In fact, both the FDA-approved hepatitis B virus and human papillomavirus vaccines are based on VLPs [20,22]. DNA vaccines are also advantageous because they induce both humoral and cellular immune responses, are easy to manufacture at large scale and at low cost, and are stable at room temperature, thus obviating the need for a cold chain for vaccine distribution and storage [23-25]. To combine the advantages of VLP and DNA-based vaccines, several studies have used a new vaccination strategy, whereby the DNA used for immunization encodes proteins allowing for formation of VLPs *in vivo*. Such DNA vaccines alone or as part of DNA prime-VLP boost vaccination strategies have been tested and shown to induce protective immune responses for various viruses, for example, hepatitis C virus [26-31], but this strategy has not been tested for ebolavirus [32-38].

Although both wild-type Ebola virus GP_1,2 _and GPΔMLD are efficiently incorporated into retrovirus particles, e.g. murine leukemia virus (MLV) [39-41], Ebola virus glycoprotein-pseudotyped VLPs based on retroviral vectors have not been explored as vaccine candidates. In this study, we tested the relative immunogenicity in mice of VLPs based on MLV, termed retrovirus-like particles (retroVLPs) bearing GPΔMLD of Ebola virus, which were generated *in vitro *(retroVLPs) or *in vivo *after injection of DNA plasmids that can produce retroVLPs *in vivo *(plasmo-retroVLP). In addition, we evaluated the immune response after immunization with plasmo-retroVLP followed by immunization with retroVLPs. For simplicity, retroVLPs and plasmo-retroVLPs are referred to as VLP and DNA, respectively, throughout the rest of this report. We compared these vaccines in mice and demonstrated that VLP, or the combination of DNA followed by VLP were both able to induce cross-species neutralizing antibody and GP_1,2_-specific IFN-γ production.

## Results

### Antigen preparation and immunization

**Figure 1:**
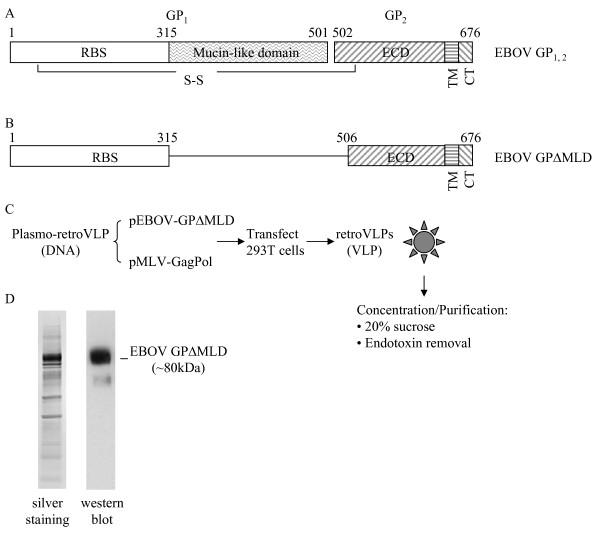
**Antigens used for immunization**. **A**. Schematic diagram of the full-length GP_1,2 _of Ebola virus (EBOV GP_1,2_, GenBank# NC_002549) that encodes a polyprotein which upon cleavage yields two subunits, GP_1 _and GP_2_, linked together through a disulfide bond. GP_1 _contains the receptor binding site (RBS) and the highly variable, highly glycosylated, and dispensable mucin-like domain (MLD). GP_2 _contains an extracellular domain (ECD), a transmembrane domain (TM) and a cytoplasmic tail (CT). The numbers above the diagram represent the amino acid residue numbers. **B**. Schematic diagram of the resulting protein expressed by plasmids used for immunization studies or to derive *in vitro *VLPs. EBOV GPΔMLD was deleted in the MLD domain and the cleavage site between GP_1 _and GP_2 _[40], so the resulting protein expressed is a single molecule. C. Production scheme for VLPs. Supernatant from HEK 293T cells containing VLPs was centrifuged through a 20% sucrose cushion to concentrate and partially purify the VLP, which was further purified to remove endotoxin. D. Verification of EBOV GPΔMLD incorporation into VLPs. The VLP product was resolved through a NuPAGE 4-12% Bis-Tris gel, the total protein was evaluated with silver staining, and EBOV GPΔMLD was detected by western blot.

VLPs were produced by transient transfection of HEK 293T cells with two plasmids: one encoding Ebola virus GPΔMLD (Figures 1A and 1B) and the other encoding the *gag-pol *polyprotein precursor of the Moloney murine leukemia virus (MLV) core and enzymatic proteins (Figure 1C). Since the retroviral vector genome was not used to produce the VLPs, there is no risk of retroviral vector-mediated genome integration associated. To maximize the yield of VLPs and their incorporation of GPΔMLD, the ratio of the two plasmids used for transfection was optimized to 2:1, *gag-pol:env *(see Materials and Methods for description; data not shown). After concentration and partial purification, the VLP preparation was checked for purity with silver stained SDS-PAGE gels and the incorporation of GPΔMLD into the VLPs was confirmed by western blot (Figure 1D). Values for residual endotoxin for VLPs, plasmids, and CpGs were 0.86 EU/ml, 0.32 EU/ml, and 0.27 EU/ml, respectively.

### Antibody response to EBOVGPΔMLD after immunization of mice

**Figure 2:**
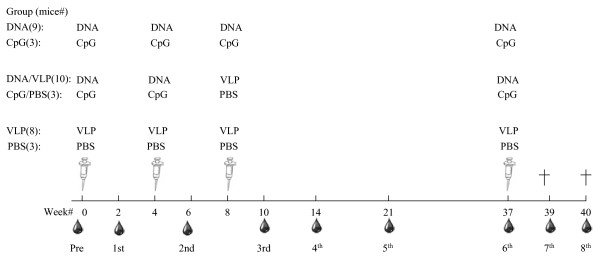
**Immunization and bleeding scheme**. Mice were divided into six groups, ranging from 3 to 10 mice per group: CpG (n = 3), CpG/PBS (n = 3) and PBS (n = 4) served as the negative controls for DNA (n = 10), DNA/VLP (n = 10) and VLP (n = 10) groups, respectively. Immunization dose and route of administration for each group is as follows: CpG (16 μg CpG/100 μl/mouse/i.m. injection), PBS (100 μl endotoxin-free PBS/mouse/i.p. injection), DNA (50 μg of pVR1012-EBOVGPΔMLD + pMLV-GagPol at a 2:1 ratio + 16 μg CpG/100 μl/mouse/i.m. injection), VLP (33 μg retroVLP/100 μl/mouse/i.p. injection). The DNA, VLP, and CpG were formulated with endotoxin-free PBS. Immunization and blood collection schedules are shown. Mice were killed (†) at week 39 and 40. The symbols of syringes and blood drops represent immunization and blood collection, respectively.

Immunization dose, routes and schedule for immunization and sample collection are depicted in Figure 2.

**Figure 3:**
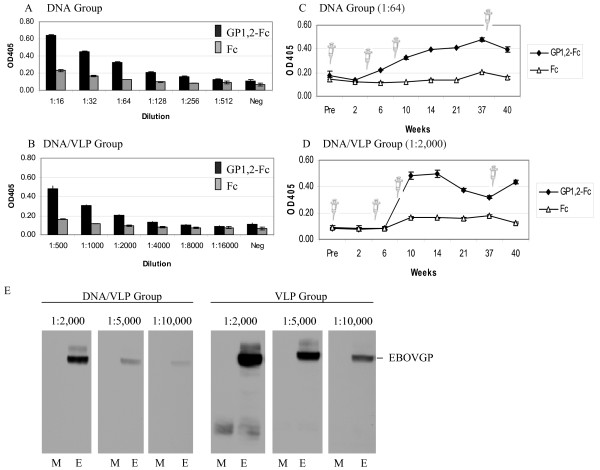
**Antibody response**. Two weeks after the third immunization, sera were collected and pooled for each group. The anti-GP_1,2 _antibody titers for the DNA group (A) and DNA/VLP group (B) were determined using a sandwich ELISA. GP_1,2_-Fc or the negative control Fc was captured to the ELISA plate using an anti-Fc antibody. Antibody titer was defined as the highest dilutions where the OD of the sample was higher than 0.15 and at least two times that of the control. The same assay was used to determine the dynamics of anti-GP_1,2 _antibodies for the DNA group (C, all diluted at 1:64) and DNA/VLP group (D, all diluted at 1:2,000). The syringe symbol indicates the time points when mice were injected with the indicated immunogen. (E) Sera from mice immunized with DNA/VLP or VLP were collected 2 weeks after the third immunization and analyzed by western blot against lysates from cells transiently expressing full-length GP_1,2 _(E, EBOV) or from cells transfected with no plasmid DNA (M, mock)

As shown in Figures 3A and 3B, the antibody titer of the sera pooled from the mice primed twice with DNA and boosted once with VLP (DNA/VLP group) was approximately 10-fold that of the mice immunized with DNA alone (DNA group) (1:2000 vs. 1:256, respectively); there was no anti-GP1,2 antibody detected in the pooled sera from either the negative control CpG, or the negative control CpG/PBS group.

To analyze the dynamics of the antibody response, we measured at different time points 1:64 dilutions of sera from the DNA group, and 1:2000 dilutions of sera from the DNA/VLP group. Figure 3C shows that the response among the DNA group climbed slowly and reached peak levels at about 14 weeks. In contrast, response of the DNA/VLP group peaked between 6-10 weeks then dipped but then responded well to a boost (Figure 3D). These data demonstrate that DNA/VLP induces a response in mice that is quantitatively and kinetically superior to that of DNA alone.

The sera collected from mice immunized with VLP alone resulted in comparable reactivity by ELISA to both the Fc control protein and the GP_1,2 _protein. Therefore, for the group of mice who were immunized with VLP alone (VLP group), the GP_1,2_-specific antibody response was evaluated by western blot from sera at the 10 week time point. Figure 3E shows that EBOV GP_1,2_-reactive antibody was detected when sera were diluted as high as 1:10,000. For comparison, sera collected at week 10 of the DNA/VLP group were reactive to EBOV GP_1,2 _at the 1:5,000 dilution, but only a faint band was detected with sera diluted to 1:10,000 (Figure 3E). The dynamics of antibody response in the VLP group was not tested because for some time points, there was insufficient serum for the western blots.

### Cross-species reactivity of the anti-EBOVGPΔMLD antibody

**Figure 4:**
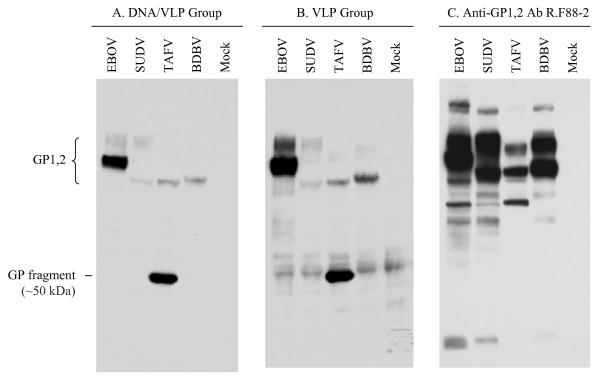
**Cross-species reactivity of anti-EBOV GPΔMLD antibodies**. Shown here are western blots using lysates from mock-transfected cells or from cells transfected with plasmids encoding full-length GP_1,2 _of EBOV, SUDV or TAFV, blotted with the pooled sera collected 2 weeks after the third immunization of the DNA/VLP group at 1:2000 dilution (**A**), the VLP group at 1:2000 dilution (**B**), or a control anti-GP_1,2 _rabbit polyclonal antibody R.F88-2 at 1:10,000 dilution [42] (**C**)

To test whether the anti-EBOV GPΔMLD antibody can recognize the full-length GP_1,2 _of other ebolaviruses, the western blot was repeated with the lysate of cells transfected with one of the following plasmids encoding the full-length GP_1,2 _of EBOV, SUDV, TAFV or BDBV. As shown in Figure 4, sera from mice immunized with DNA/VLP or VLP alone detected the full-length GP_1,2 _of all ebolaviruses tested. It was not surprising that the bands for the lysate from EBOV GP_1,2_-expressing cells were the most intense (Figures 4A and 4B), because the EBOV was the source of the immunogen, and conservation of the non-MLD domains among the ebolaviruses is not 100%. As a control for transfection efficiency, we used the rabbit anti-GP_1,2 _polyclonal antibody R.F88-2, because it was previously shown to be cross-reactive to all GP_1,2 _s used in this study [42]. Detection of similar band intensities in all lysates suggests similar levels of GP_1,2 _expression, with the exception of TAFV, which is somewhat lower (Figure 4C). Western blots using sera collected from control mice sera were negative (data not shown).

As shown in Figures 4A and 4B, a TAFV GP_1,2 _fragment of approximately 50 kDa was repeatedly detected by the immune sera but not by R.F88-2. Since R.F88-2 was raised by injecting a conserved 38-mer GP_1,2 _peptide (aa72-109, [42]), the epitope on the TAFV fragment detected by the mice presumably is from a region outside of this domain, or the region that R.F88-2 recognizes is conformationally distinct.

### Neutralization activity of the sera from immunized mice

**Figure 5:**
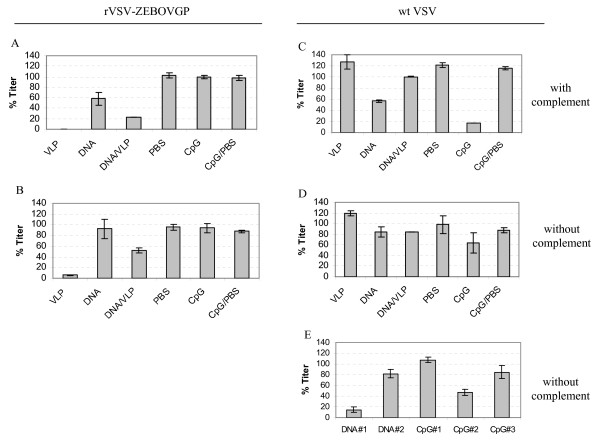
**Neutralization of rVSV-ZEBOVGP**. Either wild-type VSV or recombinant VSV with its envelope G protein replaced with EBOV GP_1,2 _[rVSV-ZEBOVGP] was pre-incubated with the pooled sera, as indicated from each group at the final concentration of 1:25 dilution in the presence (A and C) or absence (B and D) of complement. Vero cells were exposed to the virus/serum mixture, and the virus titer was quantified by counting the number of plaques. The data shown represent analyses of pooled sera collected from mice in each group at week 40: VLP (n = 5), DNA (n = 6), DNA/VLP (n = 7), or serum from single animals for each of the following groups, PBS, CpG, and CpG/PBS. E, the data represent analysis of the sera collected from the indicated individual mice at week 37 prior to the last boosting. Shown here are the average titers +/- standard deviation (SD) of the triplicate samples tested. The titer was normalized to the inoculation titer, which was calculated from transduction in the absence of any mouse serum. Representative data from two or more experiments are shown.

The neutralization activity of sera from immunized mice was first tested using recombinant vesicular stomatitis virus (rVSV) after replacing its envelope G protein with EBOV GP_1,2 _(rVSV-ZEBOVGP) [43]. In the presence or absence of complement, titers of rVSV-ZEBOVGP were reduced by 90-100% by sera from the VLP group, and by 50-80% by sera from the DNA/VLP group (Figures 5A and 5B). Whereas sera from mice immunized with DNA alone specifically neutralized rVSV-ZEBOVGP in the presence of complement (40%), no neutralization was detected in the absence of complement (Figures 5A and 5B). Surprisingly, there was an unexpected decrease in titer (40%) observed for wild-type VSV control for sera from mice immunized with DNA, but not for sera from mice immunized with DNA/VLP (Figure 5C).

It was also unexpected that within the CpG control group, one out of three mice showed neutralization activity against wild-type VSV (Figure 5C), which persisted, albeit at a lower level, in the absence of complement (Figure 5D). In addition, individual variability was observed when sera from one of two mice in the DNA group and one in three individual mice in the CpG group collected at week 37, showing reproducible neutralization activity to wild-type VSV (Figure 5E).

**Figure 6:**
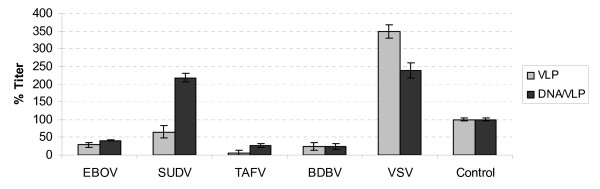
**Neutralization of MLV pseudotyped virus bearing envelopes from different viruses**. β-galactosidase-expressing MLV pseudotypes bearing the GP_1,2 _of EBOV, SUDV, TAFV, or BDBV or the G protein of VSV were pre-incubated with the pooled sera of each group (VLP (n = 5), DNA (n = 6), DNA/VLP (n = 7)), which were collected at week 40, at the final concentration of 1:25 dilution. The control group represents a pool of sera collected from one mouse in each of the negative control groups: CpG, CpG/PBS, and PBS. Vero cells were exposed to virus/antibody mixtures and the virus titer was quantified by counting the number of blue forming units (BFU) under microscope. Two or three replicates were used in each experiment. For each pseudotype, the titer observed in the presence of sera pooled from negative control mice was normalized to 100%, and all other titers for that particular pseudotype are reported as relative to that value. Representative data from two or more experiments are shown.

To confirm and extend the observed neutralization of rVSV-ZEBOVGP to other ebolaviruses, the neutralization activity of the immune sera was also tested using MLV pseudotyped with the GP_1,2 _of different ebolaviruses or the G protein of VSV as a control for specificity. As shown in Figure 6, the pooled sera of the VLP group neutralized all ebolaviruses tested by approximately 40-95% at the 1:25 dilution. No neutralization was observed against control MLV pseudotypes carrying VSV G, indicating the neutralization observed is specific. The DNA/VLP group sera neutralized the MLV pseudotypes 60-80% for those carrying the GP_1,2 _of EBOV, TAFV, or BDBV, but not the SUDV GP_1,2 _or control envelopes. The DNA group sera did not neutralize any of the MLV pseudotyped viruses tested (data not shown).

### Cellular immune response against GP_1,2_

**Figure 7:**
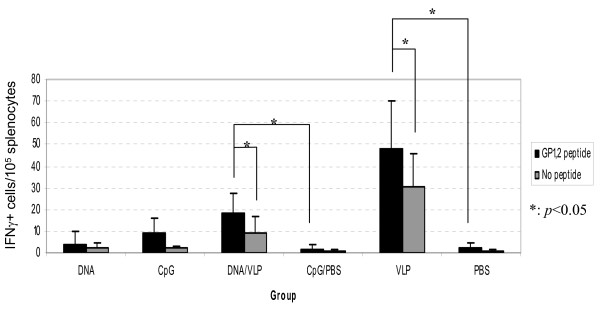
**Cellular Immune Response**. Splenocytes of each mouse in the VLP group (n = 5), DNA group (n = 6) and the DNA/VLP (n = 7) were stimulated with three EBOV GP_1,2 _peptides (ZGP-1, ZGP-4 and ZGP-5) or no peptide as a negative control. Three replicates were used for each combination. Splenocytes from a single mouse were used for each negative control group: CpG, CpG/PBS, and PBS. The numbers of IFN-γ secretion cells (spots) were counted using an ELISPOT reader (Axio Imager M2, Zeiss, Thornwood, NY). Representative data from two experiments are shown.

To assess whether immunization with VLP or DNA can induce GP_1,2_-specific cellular immune responses, splenocytes were harvested from immunized mice at week 40, and then stimulated with three GP_1,2_-specific peptides or no-peptide control, and the secretion of IFN-γ was detected by ELISPOT. Consistent with the antibody response, a small but statistically significant increase in IFN-γ-secreting cells was detected in splenocytes harvested from both the VLP and DNA/VLP groups, but not the DNA or negative control groups (Figure 7).

## Discussion

In this study we have compared three different vaccination strategies: VLPs alone, DNA that produces VLPs *in vivo*, or DNA followed by VLPs. We evaluated both the humoral immune response over time after a total of four immunizations, and the cellular immune response at week 40 upon completion of all immunizations. Using a combination of ELISA, western blot analysis, and two different types of neutralization assays, we were able to demonstrate that mice immunized with VLPs bearing the GPΔMLD of a single ebolavirus, EBOV, generated cross-reactive neutralizing antibodies to the full-length GP of EBOV, as well as of the other ebolaviruses SUDV, TAFV, and BDBV. In addition, we showed that mice immunized with VLPs bearing GPΔMLD developed a low, but detectable GP_1,2_-specific cellular immune response.

Our study did not include a challenge component, and therefore, we cannot claim that the vaccination strategy described here is protective. However, the detection of neutralizing antibodies in the range of 1:25 dilution of sera is comparable to levels of neutralizing antibodies observed in rodent and non-human primate challenge studies where protection was observed, suggesting that each of these strategies may protect against natural infection [43,44].

The observation that sera from immunized animals neutralize VSV or MLV pseudotypes carrying full-length GP_1,2_, suggests that the induced antibodies recognize full-length wild-type GP_1,2_. We had hypothesized that deleting the highly variable and highly immunogenic MLD may expose other antigenic determinants that are conserved and thus more likely to induce cross-reactive immune response. Our finding that sera from mice immunized with EBOV MLD-deleted GP_1,2 _could also cross-react with GP_1,2 _of other ebolaviruses by western blot and in some cases cross-neutralize suggests that indeed this may be the case.

The immunogenicity of the EBOV MLD-deleted GP_1,2 _has been previously studied [11,45]. Dowling et al. showed that wild-type EBOV GP_1,2_-immunized mice induced antibodies mainly against the MLD. However, 10 out 15 mice immunized with Δ12 (a GP_1,2 _in which amino acid residues 312-411 were deleted), eight out of 17 mice immunized with Δ234 (a GP_1,2 _in which amino acid residues 342-462 were deleted) and five out of 17 mice immunized with Δ1234 (a GP_1,2 _in which amino acid residues 312-462 were deleted) survived lethal challenges with mouse-adapted EBOV, suggesting the MLD is not absolutely required for inducing protective immunity. In this study, amino acid residues 316-505 of GP_1,2 _were deleted. A potentially significant difference between this study and the one by Dowling et al. is that the GPΔMLD used in this study does not contain the cleavage site between GP_1 _and GP_2_, which would help stabilize the GP_1,2 _trimer and might stimulate the immune system more efficiently. Martinez et al. reported that the MLD plays a role in stimulating dendritic cells [45]. However, antibody and cellular immune responses were not determined. GP_1,2 _was not presented in the VLP format in either of the two mentioned studies.

Each of the three vaccination approaches used in this study has its own advantages and disadvantages. The VLP group induced the strongest immune responses in terms of antibody titer and the number of IFN-γ secreting cells. However, the manufacturing of this type of vaccine is complex and costly. In addition, we observed non-specific reactivity making certain analyses of the immune response uninterpretable (i.e., ELISA could not be used). This is not surprising because retroviruses are well-documented to incorporate non-viral membrane proteins into viral particles [46-49]. The DNA group had a detectable, but low titer antibody response 2 weeks after the second immunization. Though antibody was induced in the DNA group and could be detected by ELISA, its titer was too low to neutralize either recombinant VSV or pseudotyped MLV viruses at a 1:25 dilution. Lower dilutions were not tested. The titer of the DNA group may be low due to inefficient VLP production *in vivo*. The DNA/VLP group may be an ideal choice because that VLP was used only once, thus alleviating VLP production cost and avoiding the non-specific antibody response, while retaining a strong immune response to all the ebolaviruses tested (Figure 4).

In support of greater specificity induced by the DNA/VLP regimen, there were fewer background bands in the western blots from the DNA/VLP immunized mice than from the VLP group, probably because of the incorporation of murine "self antigens" on the *in vivo *produced VLPs, compared to human 293T cell-derived antigens on in vitro produced VLPs. Therefore, *in vivo *generated VLPs from DNA immunization would not induce strong antibody response to the non-viral components of the VLP because there was only one exposure of the immune system to foreign antigens on the in vitro produced VLPs. For the VLP alone group, however, there were multiple exposures of the immune system to foreign antigens on the *in vitro *produced VLPs, and therefore, higher antibody responses to these foreign antigens were observed.

It remains to be confirmed whether the immune responses induced by the vaccination strategies described in this study can protect immunized mice from virus challenge. If so, these strategies may be adopted for other viruses whose envelope proteins include highly variable domains that shield the more conserved and potentially immunogenic regions from recognition by the immune system. By deleting the highly variable regions, the relatively conserved regions of the envelope protein will be exposed and presented to the immune system through virus like particles.

## Conclusion

Cross-species humoral and cellular immune responses were successfully induced using retrovirus-like particles (retroVLPs) bearing Ebola virus GPΔMLD. The findings suggest that GPΔMLD presented through retroVLPs may provide a strategy for development of a vaccine against multiple ebolaviruses. Similar vaccination strategies may be adopted for other viruses whose envelope proteins contain highly variable regions.

## Materials and methods

### Cell culture

Human embryonic kidney cells expressing the SV40 large T antigen (HEK 293T) (a gift from T. Dull, Cell Genesys, CA), African green monkey kidney epithelial cells (Vero cells [ATCC CCL-81]) and African green monkey kidney fibroblast-like cells expressing the SV40 T antigen (Cos7 cells [ATCC CRL-1651]) were maintained in Dulbecco's Modified Eagle's Medium (DMEM, Lonza, Walkersville, MD) supplemented with 10% heat-inactivated fetal bovine serum (FBS, HyClone, Logan, UT), 2 mM glutamine, 100 U/ml penicillin, and 100 μg/ml streptomycin (Lonza, Walkersville, MD). Cells cultures were grown at 37°C in a humidified 5% CO_2 _incubator.

### Plasmid DNAs

The pVR1012-EBOVGP, pVR1012-SUDVGP, and pVR1012-TAFVGP plasmids encode EBOV, SUDV, and TAFV GP_1,2_, respectively, and were kindly provided by Gary Nabel (Vaccine Research Center, NIH, Bethesda, MD) and Anthony Sanchez (CDC, Atlanta, GA). pVR1012-EBOVGPΔMLD encodes the EBOV MLD-deleted GP_1,2 _and was described previously [40]. pBDBV GP_1,2 _encodes wild-type BDBV GP_1,2 _and was previously described [42]. pMLV-GagPol is a Moloney murine leukemia virus (MLV)-based *gag-pol *expression plasmid and pRT43.2nlsβgal is a MLV-based packageable genome encoding β-galactosidase and a nuclear localization signal [50]. pVSV-G is a commercial plasmid from Clontech (Mountain View, CA), encoding the G glycoprotein of Vesicular stomatitis virus (VSV).

### Peptides

The following EBOV GP_1,2 _peptides were synthesized and Reverse Phase HPLC-purified at the FDA CBER Core Facility: ZGP-1 (VSGTGPCAGDFAFHK, amino acid 141-155) [51], ZGP-4 (LYDRLASTV, amino acid 161-169) and ZGP-5 (EYLFEVDNL, amino acid 231-239) [52].

### VLP and CpG production and characterization

VLPs were produced by cotransfecting 16 μg of pVR1012-EBOVGPΔMLD and 8 μg pMLV-GagPol into HEK 293T cells at a density of 5 × 10^6 ^cells/100 mm cell culture dish by using 60 μl per dish of Lipofectamine 2000 (Invitrogen, Carlsbad, CA). Supernatants were collected 48 and 72 h post transfection, clarified through 0.45 μm-pore size filters, and concentrated by Amicon Ultracel 100 k centrifugal filters (Millipore, Billerica, MA). The concentrated VLP-containing supernatants were centrifuged through a 20% (wt/vol) sucrose cushion in TNE buffer (10 mM Tris [pH 8.0], 1 mM EDTA, 100 mM NaCl) at 82,705 × *g *in a Beckman XL-90 ultracentrifuge using a Beckman SW28Ti rotor. The resulting pellets were resuspended in endotoxin-free phosphate-buffered saline (PBS) (Teknova, Hollister, CA) and stored at -80°C. A total of ten batches of VLPs were generated, pooled together and total protein concentration was measured using the Bio-Rad DC Protein Assay Kit (Bio-Rad, Hercules, CA). Western blot and silver stain analyses were performed to characterize the purity and makeup of the purified VLPs. Briefly, 10, 1, and 0.1 μg of the samples were denatured for 5 min at 95°C in 1× NuPAGE LDS Sample Buffer and 1× NuPAGE Sample Reducing Agent (Invitrogen, Carlsbad, CA), electrophoresed on a pre-cast NuPAGE 4-12% Bis-Tris gel at 200 V and transferred to a PVDF membrane (Invitrogen, Carlsbad, CA) for 90 min at 30 V. The membrane was probed with 35 ng/ml of an anti-GP_1,2 _rabbit polyclonal antibody R.F88-2, which was raised by injecting a conserved 38-mer GP_1,2 _peptide (amino acid residues 72-109) [42], followed by incubation with goat anti-rabbit IgG conjugated with horse radish peroxidase (HRP) diluted at 1:10,000 (Thermo Scientific, Rockford, IL), developed with Western Lightning™ Plus Chemiluminescence Reagent (PerkinElmer, Waltham, MA), and subsequently exposed to a Kodak BioMax MR Film (Carestream Health, Rochester, NY). Likewise, a silver stain analysis was performed using the SilverQuest™ Silver Staining Kit (Invitrogen, Carlsbad, CA) according to the manufacturer's instructions. Phosphorothioate CpG ODN 1555 (5'-GCTAGACGTTAGCGT-3', underlined portion represents the active CpG motif) was synthesized at the CBER core facility. Endotoxin was removed using the ToxinEraser™ Endotoxin Removal Kit and residual endotoxin was measured using the ToxinSensor™ Chromogenic LAL Endotoxin Assay Kit, following the manufacturers' manuals (GenScript, Piscataway, NJ).

### Animals and vaccination experiment

Female BALB/c (H-2^d^) mice, aged 6-8 weeks, were obtained from Charles River Laboratories (Germantown, MD) and divided randomly into six vaccination groups. For VLP immunizations, mice were injected intraperitoneally (i.p.) with 33 μg of VLPs in 100 μl endotoxin-free PBS. For DNA immunizations, mice were injected intramuscularly (i.m.) with a combination of 50 μg of DNA (pVR1012-EBOVGPΔMLD + pMLV-GagPol at a 2:1 ratio) and 16 μg CpG in 100 μl endotoxin-free PBS. For CpG immunizations, mice were injected with 16 μg CpG in 100 μl endotoxin-free PBS via the i.m. route. The mice were either immunized with VLPs, DNA, a combination of DNA and VLPs, CpG or endotoxin-free PBS (Figure 2). Two weeks after each injection, blood samples were collected by nicking the tails with #10 Carbon Steel Surgical Blades (Braintree Scientific, Braintree, MA) and collecting blood in BD Microtainer tubes (Becton, Dickinson and Company, Franklin Lakes, NJ). Blood was allowed to clot for 1-2 h at room temperature, centrifuged at 8,600 *xg *for 3 min, and the resulting serum in the supernatant was collected and stored at -20°C. The animal protocol and procedures were approved by Institutional Animal Care and Use Committees at the Center for Biologics Evaluation and Research (protocol #2009-04) in animal facilities accredited by the Association for Assessment and Accreditation of Laboratory Animal Care International. All experiments were performed according to institutional guidelines.

### ELISA

Immuno 96 MicroWell plates (Nunc, Rochester, NY) were coated with 50 μl of 1 μg/mL anti-human Fc IgG antibody (Kirkegaard & Perry Laboratories, Gaithersburg, MD) in PBS overnight at 4°C. The next day, plates were washed once with PBS and blocked with 100 μl of 3% Bovine Serum Albumin (BSA) in PBS for 1 h at 37°C. Plates were then incubated with 50 μl of either Fc or GP_1,2_-Fc (1 μg/ml) [43] in TBS-T (Tris-buffered saline, 0.1% Tween-20) for 90 min at 37°C. After washing 2× with TBS-T, 50 μl of each serum sample diluted in TBS-T, as indicated in the figures and legends, was added and incubated for 1 h at 37°C. Plates were then incubated with 50 μl horseradish peroxidase (HRP)-conjugated goat anti-mouse IgG diluted at 1:500 (Pierce, Rockford, IL) for 40 min at 37°C after 2 washes with TBS-T. After washing 4× with TBS-T, 100 μl/well of the ABTS substrate (Kirkegaard & Perry Laboratories, Gaithersburg, MD) was added for 3 min at room temperature and plates were read on a VICTOR^3 ^V plate reader (Perkin Elmer, Shelton, CT) at 405 nm.

### Western blot

Cell lysates for western blots were prepared by transiently transfecting 5 × 10^6 ^HEK 293T cells/100 mm cell culture dish as previously described [53] except for the following modifications: cells were transfected with 24 μg per plate of either pVR1012-EBOVGP, pVR1012-SUDVGP, pVR1012-TAFVGP or pBDBVGP. A mock transfection was also performed with no plasmid DNA. Total protein concentration in the cell lysates was measured by using the Bio-Rad DC Protein Assay Kit (Bio-Rad, Hercules, CA) and 20 μg were loaded in each lane on a pre-cast NuPAGE 4-12% Bis-Tris gel, electrophoresed, and transferred to PVDF membranes, as previously described [53]. The membranes were incubated with one of the following: pooled sera from the VLP group diluted at 1:2,000, pooled sera from the DNA/VLP group diluted at 1:2,000 or an anti-GP_1,2 _rabbit polyclonal antibody R.F88-2 diluted at 1:10,000 [53] Secondary antibody incubation was performed using HRP-conjugated goat anti-mouse IgG (1:5,000) or goat anti-rabbit IgG (1:10,000) (Thermo Scientific, Rockford, IL) and developed following the same protocol as above. Another western blot was also performed to titrate the pooled sera from the VLP and DNA/VLP groups at the dilutions of 1:2,000, 1:5,000 and 1:10,000.

### Neutralization of rVSV-ZEBOVGP

The recombinant VSV virus expressing the EBOV GP_1,2 _[rVSV-ZEBOVGP] or the wild-type VSV were generated as described previously [43]. Briefly, BSR-T7 cells were co-transfected with pBS-N, pBS-P, pBS-L, and pVSV-EBOVGP or pVSVFL(+). After 48 h of incubation at 37°C, supernatants were collected, titrated on Vero E6, and stored at -80°C. For neutralization, VeroE6 cells were seeded at 50% confluency in 6-well plates and incubated at 37°C overnight. rVSV-ZEBOVGP or wild-type VSV (100 pfu) in 45 μl of medium, which was prepared with or without 5% guinea pig complement (Accurate Chemical Corp. Westbury, NY), was mixed with mouse serum at the final concentration of 1:25 dilution and incubated overnight at 4°C. Normal mouse serum pre-diluted at 1:25 was used as the negative control. On the next day, the virus-serum mixtures were added to the cells and incubated for 1 h at 37°C. Each serum was tested in duplicate samples. After washing two times with medium, the cells were overlaid with medium containing 1% Bacto-agar and incubated at 37°C. After 48 h, the cell monolayers were fixed with 10% TCA and stained with 1% crystal violet for 30 min. Plaque numbers were used to calculate the titer.

### Neutralization of MLV pseudotyped virus bearing envelopes from different viruses

Retroviral vector pseudotypes were generated by cotransfecting HEK 293T cells as previously described [53] but modified by using the following plasmids: 10 μg of pRT43.2nlsβgal, 2.5 μg of pMLV-GagPol and 5 μg of the expression plasmid pVR1012-EBOVGP, pVR1012-SUDVGP, pVR1012-TAFVGP, pBDBVGP, or pVSV-G. One day prior to neutralization, 4 × 10^4 ^cells/well of Vero were seeded in 24-well cell culture plates. The next day, the vector pseudotypes were thawed on ice, incubated with serum at the final concentration of 1:25 dilution for 1 h at 37°C, supplemented with 8 μg/mL polybrene (American Bioanalytical, Natick, MA) and 200 μL of this mixture replaced the medium on Vero cells. After an overnight culture, supernatants were removed from the wells and replaced with 1 ml of complete culture media. 48 h after transduction, cells were fixed and histochemically stained for β-galactosidase activity, and the titer was quantified by microscopic enumeration of blue forming units (BFU), as previously described [54].

### Interferon (IFN)-γ ELISPOT assay

Erythrocytes from mice splenocytes were depleted by incubating in 1× BD PharmLyse Buffer (Becton, Dickinson and Company, Franklin Lakes, NJ) for 5 min at room temperature according to manufacturer's instructions. The splenocytes were then used in an IFN-γ ELISPOT assay as previously described [55] except for the following modifications: 500,000 splenocytes per well were stimulated with 50 μl of 2 μg/ml ZGP-1, ZGP-4, ZGP-5 or a no-peptide negative control and 0.5 μg/ml biotinylated anti-mouse IFN-γ (Clone R4-6A2) (BD Pharmingen, Franklin Lakes, NJ) was added per well in the staining process. Three replicates were used for each combination.

### Statistical analysis

To evaluate statistical significance of the ELISPOT results, we evaluated both within-group and between-group treatment difference. For the within-group comparison, three replicates for each sample were averaged and the difference between these averaged responses with respect to treatment was tested using the paired *t*-test. For the between-group comparison, the treatment difference for each mouse was obtained by taking the difference between treated vs. untreated averaged response (averaged over three replicates). This individual treatment difference was then used to compare the treatment effect between groups. Two-sample *t*-test was used for each two-group comparison.

## Competing interests

JHK performed this work as an employee of Tunnell Consulting, Inc., a subcontractor to Battelle Memorial Institute under its prime contract with NIAID, under Contract No. HHSN272200200016I.

## Authors' contributions

WO and CAW conceived and designed the experiments; WO, JD and JJ performed the experiments; JS performed statistical analysis; GP, JHK, VW, DV and GK provided materials, advice and technical assistance; WO, JD and CAW drafted the manuscript. All authors read and approved the final manuscript.
